# When Numbers Get Heavy: Is the Mental Number Line Exclusively Numerical?

**DOI:** 10.1371/journal.pone.0058381

**Published:** 2013-03-06

**Authors:** Kevin J. Holmes, Stella F. Lourenco

**Affiliations:** 1 Department of Linguistics, University of California, Berkeley, California, United States of America; 2 Department of Psychology, Emory University, Atlanta, Georgia, United States of America; University of Bath, United Kingdom

## Abstract

The *mental number line*, with its left-to-right orientation of increasing numerical values, is often regarded as evidence for a unique connection between space and number. Yet left-to-right orientation has been shown to extend to other dimensions, consistent with a general magnitude system wherein different magnitudes share neural and conceptual resources. Such observations raise a fundamental, yet relatively unexplored, question about spatial-numerical associations: What is the nature of the information represented along the mental number line? Here we show that this information is not exclusive to number, simultaneously accommodating numerical and non-numerical magnitudes. Participants completed the classic SNARC (Spatial-Numerical Association of Response Codes) task while sometimes wearing wrist weights. Weighting the left wrist–thereby linking less and more weight to right and left, respectively–worked against left-to-right orientation of number, leaving no behavioral trace of the mental number line. Our findings point to the dynamic integration of magnitude dimensions, with spatial organization instantiating representational currency (i.e., more/less relations) shared across magnitudes.

## Introduction

Since the seminal study of Dehaene, Bossini, and Giraux [1], a wealth of research has examined the so-called *mental number line* [2], [3]. Building on the original finding that Westerners respond faster to smaller numbers on the left side of space and larger numbers on the right (the Spatial-Numerical Association of Response Codes, or *SNARC*, effect), recent research points to the pervasiveness of left-to-right orientation of number, showing that numerical value biases spatial attention [4], [5] and that spatial attention influences numerical estimates [6], [7]. Although such findings could be regarded as evidence for a unique connection between space and number [2], [8], [9], left-to-right orientation has been shown to extend to other dimensions, including duration [10]–[14], physical size [15], and even emotional expression (e.g., less happy on the left, more happy on the right) [16]. Such observations converge with the notion of a *general magnitude system*, wherein different magnitudes share neural and conceptual resources [17]–[21]. Accumulating evidence–including overlapping neural activity for different magnitudes in the intraparietal sulcus (IPS) of monkeys [22] and humans [23], and reciprocal cross-magnitude interactions in human infants [24]–[26] and adults [27]–[30]–supports the existence of such a system.

That number and other magnitudes share processing mechanisms raises a fundamental, yet surprisingly underexplored, question about spatial-numerical associations: What is the nature of the information represented along the mental number line? One possibility is that this information is strictly numerical, with left and right linked exclusively to less and more numerosity, respectively [2], [8]. An alternative possibility is that the mental number line is not exclusive to number, but, instead, accommodates multiple dimensions for left-to-right orientation of magnitude relations (i.e., more/less) more generally, whether numerical or non-numerical. In other words, given that there are well-documented cognitive interactions between numerical and non-numerical magnitudes [23], [27]–[29], [31], [32] and that representations of different magnitudes share a similar organizational structure (i.e., left-to-right orientation) [1]–[7], [10]–[16], the mental number line might itself show systematic influence from other magnitudes.

We investigated these possibilities by having people make judgments about numbers while exposed to irrelevant information about another magnitude, namely weight [32], [33]. Participants completed the classic SNARC task, judging number parity (odd/even) via left- and right-side manual responses, but with a 5-lb. weight fastened to their left wrist (Left condition), right wrist (Right condition), or neither wrist (Baseline condition). Unlike previous studies examining left-to-right orientation of non-numerical magnitude stimuli, in which participants made judgments about such stimuli directly [10]–[16], the present task examined left-to-right orientation of *number*; the non-numerical magnitude information–weight–was unrelated to the task. Of particular interest was whether weight would nevertheless influence the mapping of number to space. The Baseline condition was identical to Dehaene et al.’s [1] original SNARC paradigm, and hence should elicit the typical left-to-right orientation of number, observed in numerous prior studies [2], [3]. The Right condition, with the 5-lb. weight attached to the right wrist and only an empty casing around the left wrist, effectively linked less and more weight to the left and right sides of space, respectively. Because this mapping is fully consistent with left-to-right orientation of number, we expected that this condition would elicit a left-to-right orientation (i.e., SNARC) effect of comparable degree to that in the Baseline condition. [One might also predict stronger left-to-right orientation in the Right condition than in the Baseline condition, but such a difference might be difficult to detect, given that facilitation effects are generally weaker than interference effects [27], [34] and that, beyond a certain point, reaction times cannot become any faster.].

The Left condition provided the critical test of numerical exclusivity. With the 5-lb. weight attached to the left wrist and an empty casing around the right, less and more weight were linked to the right and left sides of space, respectively–conflicting with left-to-right orientation of number, and thus allowing us to examine whether the mental number line simultaneously accommodates numerical and non-numerical magnitudes. If the mental number line is fully number-specific and independent of other magnitudes, the conflicting weight-to-space mapping introduced in the Left condition should have no impact on the spatial organization of number. However, if the mental number line instead shows influence from non-numerical magnitudes, weighting the left wrist (but not the right) should work against left-to-right orientation of number, perhaps leaving no behavioral trace of this preexisting mental mapping.

## Methods

### Ethics Statement

Study procedures were approved by the Institutional Review Board (IRB) at Emory University, Atlanta, GA. All participants provided written informed consent.

### Participants

Twenty-four undergraduates, mostly right-handed as assessed by the Edinburgh Handedness Inventory (EHI; *M*: 71.4, range: −42.9–100) [35], participated for course credit. Participants reported normal or corrected-to-normal vision.

### Materials and Procedure

**Figure 1:**
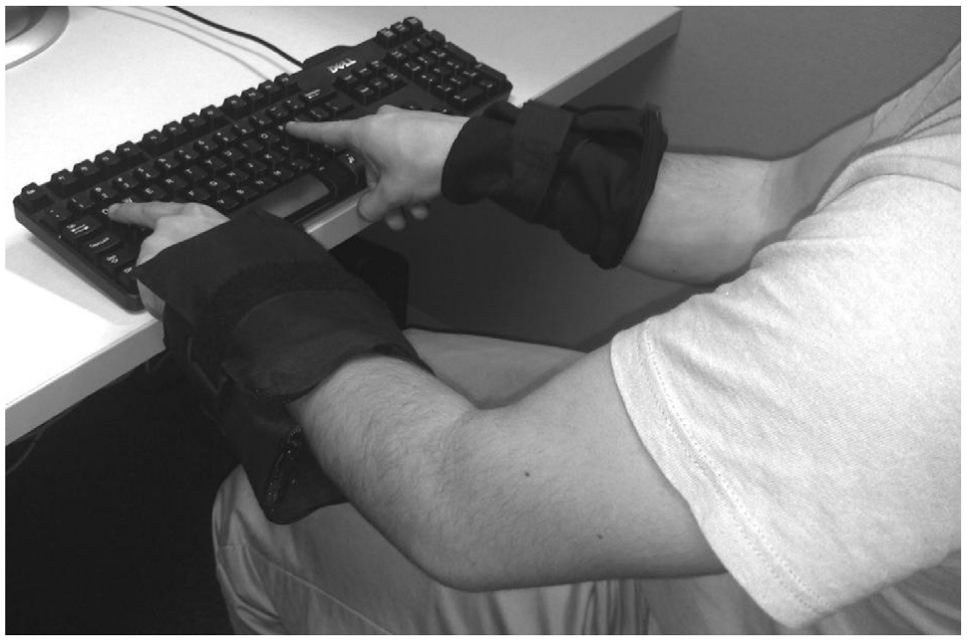
Experimental setup (shown here: Left condition). As can be seen in this photograph, the participant wore casings on both wrists. In this example, it is the left-wrist casing that contains the 5-lb. weight.

At the beginning of the experiment, the experimenter fastened nylon casings snugly around both of the participant’s wrists (see Figure 1). Each participant completed a parity judgment task under three conditions (within-subjects; order counterbalanced). In the Left and Right conditions, the experimenter attached 5-lb. weights to the left or right casing, respectively, and participants wore them for the duration of these trials. In the Baseline condition, no weights were attached, but, as in the other conditions, participants wore casings around both of their wrists. The computer keyboard was secured to the end of the table to prevent participants from resting the weights at any point during the experiment.

Each condition consisted of two blocks of trials: one in which even responses were assigned to the left key (“Q”) and odd responses to the right (“P”), and the other with the reverse assignment (order counterbalanced). There were 10 practice and 60 test trials in each block, for a total of 120 test trials in each condition. On each trial, a 500-ms fixation cross was presented centrally, followed by an Arabic numeral (0–9; Arial font; 2.9°×1.7°), which remained onscreen until participants responded (500-ms intertrial interval). Instructions emphasized speed and accuracy.

## Results

Test trials were trimmed for incorrect responses (4.9% of trials) and reaction times (RTs) greater than 2.5 standard deviations (SDs) from individual means (2.8%). Mean RT on remaining trials was 590 ms (*SD* = 105), 582 ms (*SD* = 87), and 583 ms (*SD* = 97) in Left, Right, and Baseline conditions, respectively, with no significant differences across conditions, *F*(2, 46) = .24, *p*>.7. For each participant, mean RTs were computed for left- and right-side responses separately by number pair (see also [1]), and, following previous research [36], RT difference scores (dRT: right minus left) were computed for each pair.

**Figure 2:**
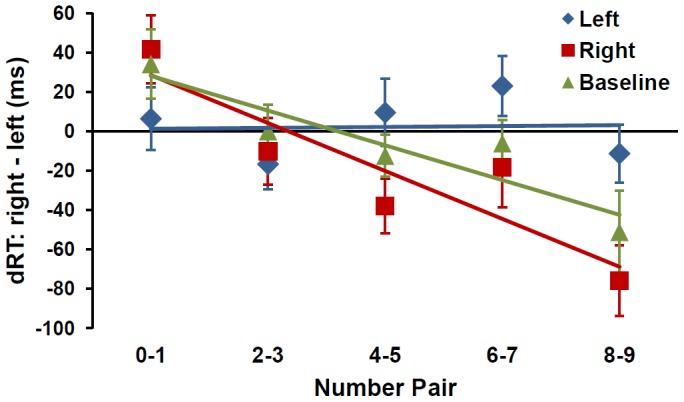
Mean reaction time difference scores (dRT) for number pairs in each condition. The Y-axis shows dRT (right RT minus left RT) scores. Negative dRT values indicate faster right-side responses, and positive dRT values indicate faster left-side responses. Error bars represent ± SEM. As the flat slope in the Left condition indicates, weighting the left wrist resulted in no consistent spatial organization of number.

dRT values were regressed on number pairs, producing unstandardized slope coefficients for each condition. A 3 (condition: Left, Right, or Baseline)×6 (order of conditions) analysis of variance (ANOVA) on slopes yielded a main effect of condition, *F*(2, 36) = 6.05, *p* = .005, but no main effect of order or interaction between condition and order (*p*s>.3). Follow-up analyses revealed that slopes in the Baseline (*M* = −9.02 ms/digit, *SD* = 12.07) and Right (*M* = −12.49 ms/digit, *SD* = 16.07) conditions differed significantly from zero (Baseline: *t*(23) = 3.66; Right: *t*(23) = 3.81; both *p*s<.002), as predicted, but not from each other (*p*>.3), indicating left-to-right orientation of number of comparable strength in the two conditions (see Figure 2). In both conditions, the majority of participants (18 of 24) responded faster to smaller numbers on the left and to larger numbers on the right (*p*s<.03, binomial tests), consistent with previous studies [2], [3], and, importantly, showing that weighting the wrist does not invariably alter spatial organization of number. In contrast, slope in the Left condition (*M* = 0.25 ms/digit, *SD* = 12.03) did not differ from zero, *t*(23) = .10, *p*>.9, indicating no consistent spatial organization (see Figure 2). Participants in the Left condition were no more likely to show left-to-right orientation of number than the reverse orientation (*p* = 1.0, binomial test). Critically, slope in the Left condition differed significantly from slopes in the other two conditions (Left vs. Baseline: *t*(23) = 2.84, *p* = .009; Left vs. Right: *t*(23) = 2.71, *p* = .01), confirming reliably weaker left-to-right orientation of number when the left wrist was weighted.

Additional analyses verified that the flat slope in the Left condition was not merely the result of slower (or faster) left-side responses to all numbers when the left wrist was weighted. The regression intercept in the Left condition (*M* = 1.06, *SD* = 78.35) did not differ from zero, *t*(23) = .07, *p*>.9, indicating that left responses (*M* = 590 ms, *SD* = 103) were no slower overall than right responses (*M* = 592 ms, *SD* = 115) in this condition. In contrast, intercepts in the Baseline (*M* = 38.00, *SD* = 60.75) and Right (*M* = 40.75, *SD* = 88.21) conditions were significantly greater than zero (Baseline: *t*(23) = 3.06; Right: *t*(23) = 2.26; both *p*s<.04), indicating faster left than right responses to smaller numbers in these conditions. We also conducted pairwise comparisons of mean RTs by side of response (left hand or right hand) across the three conditions (i.e., Left vs. Baseline, Left vs. Right, and Baseline vs. Right; for descriptive statistics, see Table 1). None of these comparisons reached statistical significance (all *p*s>.2), further suggesting that the differences in left-to-right orientation of number across conditions were not due to overall slower (or faster) manual responses, whether left- or right-side responding.

**Table 1:** Mean RT in ms (SD) by Condition and Side of Response.

	Side of Response	
Condition	Left	Right
Left	590 (103)	592 (115)
Right	593 (93)	573 (85)
Baseline	587 (97)	580 (100)

Supplemental analyses revealed that the results did not vary as a function of handedness. We conducted a median split on EHI scores to compare the performance of participants who were strongly right-handed (*M* = 92.8, *SD* = 5.9) with that of participants who were weakly right-handed (*M* = 49.9, *SD* = 36.2). [Our sample included only one true left-hander (EHI<0) and one ambidextrous participant (EHI = 0), and hence did not allow for a comparison of right-handers versus left-handers.] For both the strongly right-handed and weakly right-handed groups, slopes in all three conditions mirrored those for the full sample. Specifically, slopes differed significantly from zero in the Baseline condition, whether participants were strongly right-handed (*M* = −10.38 ms/digit, *SD* = 15.24), *t*(11) = 2.36, *p* = .04, or weakly right-handed (*M* = −7.67 ms/digit, *SD* = 8.26), *t*(11) = 3.22, *p* = .01, and in the Right condition, whether participants were strongly right-handed (*M* = −14.94 ms/digit, *SD* = 17.19), *t*(11) = 3.01, *p* = .01, or weakly right-handed (*M* = −10.03, *SD* = 15.21), *t*(11) = 2.28, *p* = .04. In contrast, slopes did not differ significantly from zero in the Left condition for either group (strongly right-handed: *M* = 0.00 ms/digit, *SD* = 10.76; weakly right-handed: *M* = 0.50 ms/digit, *SD* = 13.66; both *p*s>.9). Moreover, in all three conditions, slopes for the two groups did not differ significantly from each other (*p*s>.4), indicating no reliable differences based on handedness.

## Discussion

Our findings suggest that left-to-right orientation of number may reflect spatial organization of magnitude more generally. By showing that weighting the left wrist effectively nullifies left-to-right orientation of number, we provide evidence that the mental number line is modulated by information from another magnitude (weight), even when such information is entirely task-irrelevant. An intriguing possibility is that this interaction between numerical and non-numerical magnitudes may occur at a relatively fine-grained level. For example, larger differences in the relative weight associated with the left and right sides of space (e.g., 5 lb. on the left, 0 lb. on the right, as in the present study) might be expected to have a stronger influence on the mental number line than smaller differences (e.g., 3 lb. on the left, 2 lb. on the right), though the latter should still weaken left-to-right orientation relative to baseline (provided that the weight differences are apprehended). Such a manipulation has the potential to provide considerable insight into how different types of magnitude information are mentally combined.

Another important issue for future research is whether the mental number line fully integrates numerical and non-numerical magnitudes or, instead, retains some specificity to number. That is, our findings might reflect a single spatial representation of magnitude, with number and weight treated relatively interchangeably (a “mental magnitude line”), or separate representations for each magnitude, with the weight-to-space mapping distinct from, yet exerting an influence on, the mental number line (see [16] for discussion). Even if the mental number line proves to be largely number-specific, our findings nevertheless lend support to the idea that numerical representations show functional interactions with representations of other magnitudes [23], [27]–[29], [31], [32], [37], [38], including their spatial format.

Although weight served as our key experimental manipulation, our findings could reflect the interaction of number with some other magnitude-related dimension, rather than with weight per se. Weighting the wrist may have introduced asymmetries in other non-numerical magnitudes, which in turn came to be associated with left and right sides of space. When the left wrist was weighted, for example, it is possible that participants perceived left-side actions as more effortful (see [39] for discussion) or associated the left side with larger objects, given that the weighted casing was somewhat bulkier than the unweighted casing (see Figure 1). Importantly, however, such possibilities are fully compatible with our conclusion that the mental number line is not exclusively numerical, accommodating both numerical and non-numerical magnitudes. Indeed, our findings might reflect the interaction of number with multiple non-numerical magnitudes that are, at least to some extent, mentally undifferentiated [23], [27]–[29], [31], [32], [37], [38].

Previous research has highlighted the processing consequences resulting from incongruity between different magnitudes (e.g., slower magnitude judgments when the numbers 3 and 5 are large and small in physical size, respectively, than vice versa [27]). Our findings add to this literature in showing that such cross-magnitude interactions can also occur when different magnitudes map differently to space, pointing to consequences for the underlying representations. We suggest that, in the case of number, the default left-to-right representation undergoes systematic change from other magnitudes. Such flexibility is consistent with evidence that factors such as reading direction [40], counting habits [41], and explicit visualization [42] likewise alter the spatial organization of number, and that spatial organization for other magnitudes may be similarly malleable [16]. That participants in the present study showed reliable left-to-right orientation of number when weight information aligned with the default representation, yet no such orientation when weight conflicted, points to the dynamic integration of magnitude dimensions. Given that weight information was presented haptically but numerical information visually, such integration may spontaneously occur across sensory modalities, consistent with proposals that underlying representations of magnitude are multimodal [19], [43], [44]. Our findings suggest that spatial organization instantiates more-versus-less relations shared across magnitudes, regardless of the myriad of forms in which they present themselves.
